# Catheter-related bloodstream infection associated with multiple insertions of the peripherally inserted central catheter in patients with hematological disorders

**DOI:** 10.1038/s41598-021-91749-4

**Published:** 2021-06-09

**Authors:** Yoshinori Hashimoto, Rina Hosoda, Hiromi Omura, Takayuki Tanaka

**Affiliations:** Department of Hematology, Tottori Prefectural Central Hospital, 730 ezu, Tottori City, Tottori 680-0901 Japan

**Keywords:** Medical research, Oncology, Risk factors

## Abstract

Patients with hematological disorders are treated with multiple cycles of chemotherapy. As a result, they often require multiple insertions of the peripherally inserted central catheter (PICC) for prolonged periods of time. Although PICCs have been widely used worldwide in various patients, the safety and feasibility of the multiple insertions of the PICC in this population have not been fully verified. We performed a retrospective analysis to clarify the relationship between complications and multiple PICC insertions in patients with hematological disorders who were treated with either chemotherapy or immunotherapy. A total of 651 PICCs were inserted in 261 patients with a median age of 66 years. Acute myeloid leukemia (AML) and non-Hodgkin's lymphoma were the most common diseases in our patient cohort. The total catheter days (CDs) was 29,485 days, with a median catheter duration of 30 days. The rate of catheter-related bloodstream infection (CRBSI) in our patient cohort at high rate of re-insertion was 2.0/1000 CDs. Although multiple PICC insertions were not a risk factor of CRBSI, our findings suggest that a prolonged catheter dwell time may be associated with CRBSI. AML was an important risk factor of CRBSI. While the PICC dwell time depends on the treatment cycle, our findings indicate that it should be limited to approximately 30 days and catheters may be removed and re-inserted as needed.

## Introduction

Insertion of a central venous catheter (CVC) is a critical procedure for patients with hematological disorders as they require frequent blood sampling as well as mid-to-long-term chemotherapy, transfusion, antimicrobial therapy, and hematopoietic stem cells transplants. Conventionally, CVCs are inserted through the internal jugular vein or subclavian vein. However, these procedures are associated with pain and fear in patients, as well as severe adverse events^1,2^. Peripherally inserted central catheters (PICCs) became available in the 1990s in Europe and the United States as a safer option for patients who require mid-to-long-term vascular access^3^. In patients with hematological disorders, PICCs are considered safer than CVCs in terms of the risk of catheter-related complications^4–7^. However, thrombosis and bloodstream infection (BSI) are major risks of PICCs, and patients with hematological disorders undergoing chemotherapy are generally considered at higher risk of these complications^8–11^. Studies also demonstrated in a cohort of patients including those with non-hematological disorders that a prolonged PICC dwell time and multiple insertions are risk factors of BSI^12,13^. Patients with hematological disorders are treated with multiple cycles of chemotherapy. As a result, they often require multiple insertions of the PICC for prolonged periods of time. Although PICCs has been widely used worldwide in various patients^14,15^, the safety and feasibility of the multiple insertions of the PICC in this population have not been fully verified. At our institution, an increasing number of patients prefer to have catheters removed and re-inserted for each treatment cycle. Thus, we performed a retrospective analysis to clarify the relationship between complications and multiple PICC insertions in patients with hematological disorders who were treated with either chemotherapy or immunotherapy.

## Patients and methods

Consecutive patients who had a PICC inserted and subsequently underwent chemotherapy or immunotherapy at our institution between November 2013 and March 2020 were included in the study. The PICCs were inserted by trained physicians, including residents who were trained by their supervisors. Groshong^®^ NTX 4 Fr. Single-Lumen Catheter, BD, USA were inserted using maximal sterile barrier precautions. All insertions were performed under ultrasound or fluoroscopic guidance, and the PICC was placed in the upper right or left arm. Patients were excluded from the study if the catheter was inserted at the bedside without fluoroscopy guidance, or if the tip of the catheter was not placed in the lower superior vena cava. Medical records were reviewed to collect information on patient characteristics, reasons for catheterization, side of catheterization, catheter length, complications during or after catheterization, reasons for catheter removal, and incidence of catheter-related bloodstream infection (CRBSI). Each case of the PICC insertion was categorized based on the number of insertions the patient has had (1, 2, and 3 + insertions), and the clinical characteristics and outcomes were compared among the groups (1 vs. 2, 2 vs. 3 + insertions) to determine the impact of multiple insertions. In order to compare single and multiple insertions, and to verify the impact of increasing the number of insertions, we used this categorization. The study data were anonymized, and the patients were assured that participation was voluntary and that they may opt out of information disclosure. The study was performed in accordance with the Declaration of Helsinki and Ethical Guidelines for Medical and Health Research Involving Human Subjects, and was approved by the ethics committee of Tottori Prefectural Central Hospital (IRB#202018), which waived the requirement for informed consent as the data were analyzed anonymously and the patient privacy was protected. A sample size calculation was not performed since the study was retrospective in nature and the samples represent continuous cases of patients who required PICC insertions for the purpose of their treatments.

### Definition

BSI is defined as either CRBSI or central line-associated BSI. In the present study—which was not designed as a surveillance study—the definition of CRBSI in the clinical sense was used. The diagnostic criteria for CRBSI were based on the "Clinical Practice Guidelines for the Diagnosis and Management of Intravascular Catheter-related Infection,” revised by the Infectious Disease Society of America in 2009^16^. More specifically, a definitive diagnosis of CRBSI was made when the same microorganisms were isolated from at least one set of cultures from blood collected from the skin and a culture of the catheter tip, or when two blood culture samples (one from the catheter hub and the other from a peripheral vein) met the criteria for CRBSI (quantitative blood culture results or the differential time to positivity [DTP], defined as the difference in the time to positivity between blood cultures). Based on quantitative blood cultures, CRBSI was diagnosed when the microbial colony count for the catheter-drawn blood sample was at least three times higher than that for the peripheral blood sample. Based on DTP, CRBSI was diagnosed when the time to positivity of the catheter-drawn blood sample was at least two hours shorter than that of the peripheral blood sample.

### Statistical analysis

Descriptive statistics were reported for the study cohort. For the comparison of the number of PICC insertions, categoric variables were compared using Fisher’s exact test and continuous variables were compared using Mann–Whitney U test. Univariate and multivariate logistic regression analyses were performed to identify risk factors of CRBSI. A two-tailed test was performed for all variables, and p < 0.05 was considered statistically significant. All statistical analyses were performed with EZR (Saitama Medical Center, Jichi Medical University, Saitama, Japan), which is a graphical user interface for R (The R Foundation for Statistical Computing, Vienna, Austria). More precisely, it is a modified version of R commander designed to add statistical functions frequently used in biostatistics^17^.

### Ethics approval and consent to participate

The study data were anonymized, and the patients were assured that participation was voluntary and that they may opt out of information disclosure. The study was performed in accordance with the Declaration of Helsinki and Ethical Guidelines for Medical and Health Research Involving Human Subjects, and was approved by the ethics committee of Tottori Prefectural Central Hospital (IRB#202018), which waived the requirement for informed consent as the data were analyzed anonymously and the patient privacy was protected.

## Results

After excluding 25 PICCs from the analysis based on the criteria (Three patients were inserted at the bedside without fluoroscopy guidance, and the tip of the catheter was not placed in the lower superior vena cava in 22 patients), a total of 651 PICCs in 261 patients with hematological disorders who underwent chemotherapy or immunotherapy were included in the study. A PICC was inserted once in 130 patients, and at least twice in 131 patients. The median frequency of the PICC insertion per patient was two, with a maximum of 29. Table 1 summarizes the characteristics of patients who had PICC insertion once (group A) or at least twice (group B). The median age of the entire cohort was 66.0 years; group B was significantly younger compared with group A. A total of 263 (40.4%) and 388 (59.6%) PICCs were placed in female and male patients, respectively. The median body surface area for the entire cohort, group A, and group B were 1.60, 1.59, and 1.60 m^2^, respectively, with no statistical difference between groups A and B. Acute myeloid leukemia (AML) and non-Hodgkin's lymphoma (NHL) were the most common diseases in the entire patient cohort. AML was more common in group B, while NHL was more common in group A. The total catheter days (CDs) was 29,485 days, with a median catheter duration of 30 days. The median catheter duration was significantly lower in group B compared with group A.

**Table 1:** Patient characteristics of entire cohort and each PICC insertion group.

Patient characteristics	Entire cohort	1st PICC group (A)	2nd or more PICC group (B)	P value A vs. B
Number of the PICCs	651	261	390	
Age, median (range)	66.0 (17–88)	67.0 (17–88)	65.0 (22–86)	0.003
**Gender**				
Female, n (%)	263 (40.4)	106 (40.6)	157 (40.3)	0.935
Male, n (%)	388 (59.6)	155 (59.4)	233 (59.7)	
BSA, median; m^2^ (range)	1.60 (0.99–2.15)	1.59 (0.99–2.15)	1.60 (1.12–2.14)	0.545
**Diseases**				
AML, n (%)	266 (40.9)	83 (31.8)	183 (46.9)	< 0.001
MDS, n (%)	33 (5.1)	10 (3.8)	23 (5.9)	0.277
ALL, n (%)	31 (4.8)	10 (3.8)	21 (5.4)	0.454
NHL, n (%)	267 (41.0)	121 (46.4)	146 (37.4)	0.028
HL, n (%)	7 (1.1)	4 (1.5)	3 (0.8)	0.447
MM, n (%)	30 (4.6)	17 (6.5)	13 (3.3)	0.084
Aplastic anemia, n (%)	7 (1.1)	7 (2.7)	0	0.002
Others, n (%)	10 (1.5)	9 (3.4)	1 (0.3)	0.002
Total catheter days	29,485	15,401	14,084	
Indwelling catheter days, median (range)	30 (2–311)	39 (3–311)	27 (2–277)	< 0.001

*ALL* acute lymphoblastic leukemia, *AML* acute myeloid leukemia, *BSA* body surface area, *HL* Hodgkin lymphoma, *MDS* myelodysplastic syndromes, *MM* multiple myeloma, *NHL* non-Hodgkin lymphoma, *PICC* peripherally inserted central catheter.

Table 2 summarizes the reasons for catheter removal and the incidence of CRBSI in the entire cohort as well as in groups A and B. In the entire cohort, most patients had their catheter removed as their treatment completion (n = 463, 71.1%). Death was the reason for catheter removal in 41 cases (6.3%). Other reasons for catheter removal included catheter-related complication (n = 120, 18.4%), infection (n = 35, 5.4%), fever (n = 22, 3.4%), occlusion (n = 19, 2.9%), accidental removal (n = 10, 1.5%), pain (n = 9, 1.4%), and thrombosis (n = 8, 1.2%). The platelet count at the time of the PICC insertion was not examined. None of the patients had severe injury as a direct result of catheter insertion. CRBSI occurred in 60 cases at a rate of 2.0/1000 CDs. Even after detection of CRBSI, some patients continued treatment without removal of the PICC, depending on the pathogenic bacteria, patient condition, and responses to antimicrobial agents. Compared with group A, removal and re-insertion of catheters were more common in patients in group B as they underwent multiple treatment cycles. As a result, most patients in group B had their catheters removed at the end of the treatment. Complication leading to catheter removal was uncommon in group B as most patients likely had their catheters removed before any complications occurred. Although there were more cases of CRBSI in group B compared with group A, there was no significant difference in the incidence of CRBSI between the two groups on univariate analysis (p = 0.273).

**Table 2:** Reasons for PICC removal and incidence of complications.

Reasons and complication	Entire cohort	1st PICC group (A)	2nd or more PICC group (B)	P value A vs. B
Number of the PICCs	651	261	390	
**Reasons**				
Treatment completion, n (%)	463 (71.1)	166 (63.6)	297 (76.2)	0.001
Death, n (%)	41 (6.3)	22 (8.4)	19 (4.9)	0.072
Transfer to another hospital, n (%)	12 (1.8)	7 (2.7)	5 (1.3)	0.238
In use, n (%)	15 (2.3)	7 (2.7)	8 (2.1)	0.604
Removal due to complication, n (%)	120 (18.4)	59 (22.6)	61 (15.6)	0.030
Clinical suspicion of infection, n (%)	35 (5.4)	13 (5.0)	22 (5.6)	0.860
Fever of unknown origin, n (%)	22 (3.4)	7 (2.7)	15 (3.8)	0.367
Occlusion, n (%)	19 (2.9)	11 (4.2)	8 (2.1)	0.152
Accidental removal, n (%)	10 (1.5)	9 (3.4)	1 (0.3)	0.002
Pain (including phlebitis), n (%)	9 (1.4)	6 (2.3)	3 (0.8)	0.167
Thrombosis, n (%)	8 (1.2)	6 (2.3)	2 (0.5)	0.065
Others, n (%)	17 (2.6)	7 (2.7)	10 (2.6)	1.000
**Complication**				
CRBSI*, n (%)	60 (9.2)	20 (7.7)	40 (10.3)	0.273
CRBSI per 1000 catheter days	2.0	1.3	2.8	

*CRBSI* catheter-related bloodstream infection, *PICC* peripherally inserted central catheter.

*Even after detection of CRBSI, some patients continued treatment without removal of the PICC, depending on the pathogenic bacteria, patient condition, and responses to antimicrobial agents.

Next, we categorized PICC insertion cases into those of second insertion (group C) and third or more insertions (group D) to examine the safety of multiple insertions in terms of the risk of complications (Table 3). Between groups C and D, there was no significant difference in patient characteristics including age and disease type. Reasons for catheter removal and the frequency of complication-associated catheter removal were also similar between the two groups. Group C has more cases of CRBSI compared with group D, and the increase in number of catheter insertions was not associated with the risk of CRBSI and complication-associated catheter removal.

**Table 3:** Comparison of the PICC groups according to insertion frequency.

Patient characteristics	2nd PICC group (C)	3rd or more PICC group (D)	P value C vs. D
Number of the PICCs	131	259	
Age, median (range)	65.0 (22–86)	65.0 (22–83)	0.677
**Gender**			
Female, n (%)	51 (38.9)	106 (40.9)	0.744
Male, n (%)	80 (61.1)	153 (59.1)	
BSA, median; m^2^ (range)	1.61 (1.12–2.09)	1.59 (1.15–2.14)	0.857
**Diseases**			
AML, n (%)	58 (44.3)	125 (48.3)	0.519
NHL, n (%)	51 (38.9)	95 (36.7)	0.740
Total catheter days	5425	8659	
Indwelling catheter days, median (range)	28.0 (3–277)	27.0 (2–199)	0.159
**Reasons for PICC removal**			
Treatment completion, n (%)	93 (71.0)	204 (78.8)	0.102
Death, n (%)	6 (4.6)	13 (5.0)	1.000
Removal due to complication, n (%)	27 (20.6)	34 (13.1)	0.076
Occlusion, n (%)	2 (1.5)	6 (2.3)	0.723
CRBSI*, n (%)	19 (14.5)	21 (8.1)	0.054
CRBSI per 1000 catheter days	3.5	2.4	

*AML* acute myeloid leukemia, *BSA* body surface area, *CRBSI* catheter-related bloodstream infection, *NHL* non-Hodgkin lymphoma, *PICC* peripherally inserted central catheter.

*Even after detection of CRBSI, some patients continued treatment without removal of the PICC, depending on the pathogenic bacteria, patient condition, and responses to antimicrobial agents.

Lastly, risk factors of CRBSI, which is the most severe complication of the PICC, were examined (Table 4). As described above, there was no association between CRBSI and multiple PICC insertions on univariate analysis. AML and a prolonged PICC dwell time (≥ 30 days) were identified as risk factors of CRBSI. NHL was identified as a negative risk factor of CRBSI. On multivariate analysis, AML remained a significant risk factor of CRBSI. There was no association between CRBSI and multiple PICC insertions on multivariate analysis.

**Table 4:** Univariate and multivariate logistic regression analyses of risk factors for CRBSI.

Variables	Univariate			Multivariate		
	OR	95% CI	P value	OR	95% CI	P value
Age ≥ 60 years	1.110	0.620–1.970	0.734			
Gender (male)	0.754	0.443–1.290	0.300			
BSA ≥ median (1.60 m^2^)	0.843	0.495–1.440	0.529			
AML	3.820	2.150–6.800	< 0.001	3.450	1.910–6.250	< 0.001
NHL	0.260	0.129–0.522	< 0.001			
Indwelling catheter days ≥ median (30 days)	1.850	1.060–3.220	0.030	1.550	0.869–2.750	0.133
Multiple insertions	1.380	0.786–2.410	0.264	1.210	0.673–2.170	0.527

*AML* acute myeloid leukemia, *BSA* body surface area, *CI* confidence interval, *CRBSI* catheter-related bloodstream infection, *NHL* non-Hodgkin lymphoma, *OR* odds ratio.

## Discussion

To our knowledge, this is the first study to examine the safety of multiple PICC insertions in a compromised host, specifically patients with hematological disorders who had undergone chemotherapy or immunotherapy. We demonstrated that the rate of CRBSI in these patients who are at high rate of the PICC re-insertion was relatively low, and multiple PICC insertions was not a risk factor of CRBSI.

There are few reports of the PICC in patients with hematological disorders^2,4–7,18–21^. Most studies are single-institution studies and demonstrate that PICCs are safer than CVCs in terms of the risk of catheter-related complications and CRBSI. While the evidence is limited, some studies demonstrated that the use of polyurethane catheters in allogeneic hematopoietic stem cell transplant recipients^20^ and acute leukemia in adults are risk factors of CRBSI^21^. Similarly, our findings also suggest that patients with progressive diseases may be at a higher risk for CRBSI. Previous studies included cases in which a PICC was inserted multiple times; however, the proportion of such cases in the entire study cohorts was limited to approximately 20%^4–7,18–21^, and the studies did not examine the direct impact of multiple catheter insertions on CRBSI risks. In our study, about 60% (390/651) of all PICC insertion cases were of multiple insertions.

There are several studies that examined risk factors of the PICC-related BSI in various populations not specific to those with hematological disorders. For example, a study in adult patients (162 PICCs, 162 patients; BSI rate, 3.13/1000 CDs) demonstrated that congestive heart failure, intra-abdominal perforation, *Clostridium difficile* infection, recent chemotherapy, presence of tracheostomy, and use of multi-lumen catheters are risk factors of BSI^22^. Similarly, another study (966 PICCs, 747 patients; BSI rate, 2.16/1000 CDs) identified hospital length of stay, intensive care unit (ICU) status, and number of device lumens as risk factors^23^. A study on hospitalized children (2592 PICCs, 1819 patients; BSI rate, 2.58/1000 CDs) identified ICU exposure, as well as administration of parental nutrition, and a prolonged catheter dwell time as risk factors^12^. Moreover, a recent prospective study (1215 PICCs, 1017 patients; BSI rate, 1.69/1000 CDs) demonstrated that previous PICC insertion was a risk factor for BSI^13^. Unlike patients with hematological disorders, only about 10% of the patient cohorts in these studies were those who underwent chemotherapy. Furthermore, these studies include a limited number of cases with multiple catheter insertions. Although these studies are limited to the comparison of BSI and non-BSI cases, they collectively indicate that a prolonged catheter dwell time and previous PICC insertion are risk factors of BSI. This is a major concern for patients with hematological disorders who require multiple insertions or long-term placement of the PICC. We performed our study in patients with hematological disorders and demonstrated the number of catheter insertions was not associated with the risk of CRBSI that although CRBSI occurred more frequently in multiple insertions. On univariate analysis, a prolonged catheter dwell time was identified as a risk factor for CRBSI. It is likely that patients who had more than one PICC insertion required catheter removal due to CRBSI but subsequently had their catheters re-inserted for the purpose of treatment. This could elevate the incidence of CRBSI in the multiple insertion group. As seen by the low incidence of CRBSI in patients with lymphoma, the risk of complications may be lower in patients with planned catheter insertions and removals. However, autopsy studies suggest that a prolonged PICC dwell time and multiple insertions may cause direct changes to the veins, including inflammation and vascular injury^24^. Furthermore, repeated PICC insertions were shown to complicate re-insertions and increase the risk of complications in children^25^. Thus, it is important to consider the impact of multiple PICC insertions.

It would be ideal to compare the outcomes of treatment cycles completed with one indwelling PICC and with the removal and re-insertion of catheters in between treatment cycles. However, a single PICC is used in most cases unless complications develop and treatment needs to be discontinued. Thus, it is challenging to discriminate cases in which PICC was intentionally removed and re-inserted from cases in which PICC was initially removed due to complications and re-inserted to continue treatment. In order to address this issue, a prospective study is needed to compare the outcomes of patients with an indwelling PICC and patients with intentional PICC removal and re-insertion for the entire duration of the treatment cycle.

## Conclusion

We demonstrated that the rate of CRBSI in immunocompromised patients who are at high rate of the PICC re-insertion was relatively low, occurring in 2.0/1000 CDs. Multiple PICC insertions was not a risk factor of CRBSI. A prolonged catheter dwell time may be associated with CRBSI, and AML was an important risk factor of CRBSI. While the PICC dwell time depends on the treatment cycle, our findings indicate that it should be limited to approximately 30 days and catheters may be removed and re-inserted as needed.
